# Supplementary material for: The contribution of ancient admixture to reproductive isolation between European sea bass lineages
Source: Evol Lett. 2020 Apr 15;4(3):226–42. doi: 10.1002/evl3.169 (PMC7293100; doi:10.1002/evl3.169)
Supplement: Supplementary file 1 — Supplementary Table 1. Summary statistics of sequencing and mapping data for each individual. Supplementary Figure 1. Depth of coverage per individual. Supplementary Figure 2. Principal Component Analysis of the European sea bass population genetic structure. Supplementary Figure 3. Statistics measured in non‐overlapping 50 kb windows along the genome. Supplementary Figure 4. Genome‐wide correlations between pairs of statistics measured in non‐overlapping 50 kb windows. Supplementary Figure 5. Introgressed tract length distributions. Supplementary Figure 6. Data and results for the SNPs and 50 kb window based HMM approach to identify regions involved in reproductive isolation between the two lineages of D. labrax along chromosome 7. Supplementary Figure 7. Distributions and joint allele‐frequency spectrums of derived D. punctatus alleles present in D. labrax. Supplementary Figure 8. Distributions of the fraction of archaic tracts for Atlantic and Mediterranean genomes inside and outside regions involved in RI. Supplementary Table 2. Values used to estimate T admix for each chromosome. [file EVL3-4-226-s001.pdf]

Supplementary Materials

Duranton et al.

The contribution of ancient admixture to reproductive isolation between European sea bass lineages

December 2019

1 - Whole-genome resequencing 2

2 – Population structure 5

3 – Test for gene flow between *D. labrax* and a third lineage 6

4 - Detection of introgressed haplotypes 8

5 - Delineation of regions involved in reproductive isolation between *D. labrax* lineages 9

6 – Frequency of introgressed *D. punctatus* tracts 10

7 – Estimation of the time since introgression between *D. punctatus* and *D. labrax* 12

8 – References 13

1 - Whole-genome resequencing

Individual	Library kit	Run	Unpaired reads examined	Reads pairs examined	Unmapped reads	Read pair duplicates	Percent duplication	Estimated library size
**AT_205899**	PCR-free	2	710525	51574949	1779055	5243750	0.102331	338339229
		1	1599933	51480924	2756181	13676012	0.26773	89393199
**AT_206125**	PCR-free	2	1162366	66285637	2548540	5600109	0.085594	533205434
		1	2099730	70022633	3645908	17135878	0.246806	135168917
**AT_205638**	PCR-free	2	592020	42345534	1467942	5222111	0.123944	225073296
		1	949465	40386831	1834665	12051209	0.299717	60298353
**AT_211069**	PCR-free	2	340601	23707392	825243	2554580	0.108193	145663521
		1	503439	21859655	978983	5980255	0.274728	36599828
**AT_211267**	PCR-free	2	718656	50229040	1771632	4354008	0.087411	386708710
		1	1676865	52761066	2870845	12284403	0.234926	107357281
**AT_207193**	PCR-free	2	933708	59620496	2174352	6025969	0.101973	396946010
		1	1725667	62518162	3095545	16425410	0.26464	109956484
AT_205057	Nano	1	847273	65506340	2196319	3561652	0.055356	770163673
AT_103278	Nano	1	818074	58633597	2032366	3419072	0.059535	634069837
AT_095165	Nano	1	569451	50971720	1623871	2381592	0.047552	676857516
AT_208678	Nano	1	783941	59577971	2004383	3017378	0.051526	754343749
NEM_07	PCR-free	2	1698446	117368673	4077408	18711889	0.160839	399832566
		1	710006	52729283	1759686	9029507	0.172299	160945863
NEM_09	PCR-free	2	1050548	83890852	2741562	10541945	0.126624	405629701
		1	480858	41381960	1304016	8080455	0.195879	117595746
NEM_10	PCR-free	2	1316276	94951027	3241172	12858549	0.136506	424680397
		1	471446	38196025	1226054	6943165	0.182347	113906586
NEM_14	PCR-free	1	1627807	92941773	3483253	13813625	0.149824	408915958
NEM_16	PCR-free	2	593154	44861040	1513572	4786524	0.107339	258646208
		1	224528	19291930	613914	3058638	0.158891	69285554
NEM_30	Nano	1	863049	68382690	2267473	3819091	0.056962	788549675
NEM_02	Nano	1	926545	70986840	2365455	4528848	0.065048	712885179
NEM_04	Nano	1	743971	64687003	2096245	3486045	0.054803	763144409
NEM_05	Nano	1	759149	59477335	1973059	2899192	0.049731	779637467
NEM_08	Nano	1	1035595	82073920	2694179	5626675	0.069673	721645856
SEM_02	PCR-free	2	465634	34841686	1192898	4342291	0.125164	169755455
		1	214257	17618667	574709	3265401	0.18572	51785382
SEM_05	PCR-free	2	928763	69243971	2348789	8962764	0.130297	323138092
		1	405300	32921159	1070004	6303847	0.192022	92016456
SEM_09	PCR-free	1	631808	46758401	1598742	6363989	0.136711	219184320
SEM_15	PCR-free	1	661396	47118796	1624458	6575007	0.140163	218120903
SEM_21	PCR-free	1	570375	40958914	1403661	6581858	0.161225	158247672
SEM_10	Nano	1	687721	60932741	1962181	3344853	0.056067	668105655
SEM_06	Nano	1	747676	63988648	2077118	4195923	0.066478	588179698
SEM_03	Nano	1	838812	67601876	2244974	4340684	0.065364	639954765
SEM_11	Nano	1	622507	60102973	1868897	4606246	0.077472	461536800
WME_78204	PCR-free	2	809260	34759734	1527580	5125749	0.148367	131097459
		1	258012	13636123	536218	2139774	0.158096	47348614
WME_97522	PCR-free	1	1057906	74398643	2547664	11626900	0.157196	259519641
		2	334180	28357701	894368	4341746	0.153585	98230713

WM_098394	Nano	1	913210	66517786	2295822	4729605	0.072642	588581722
WM_098577	Nano	1	998106	67307402	2403470	4393065	0.066926	665052074
WM_101174	Nano	1	661295	55575994	1816347	2859239	0.052294	687069162
WM_102209	Nano	1	958027	70934697	2430433	4794683	0.068752	674026270
WM_095636	Nano	1	832945	65205207	2218543	4450311	0.069881	603852551
WM_103016	Nano	1	936302	74935945	2439552	5067344	0.068693	698973985
WM_204910	Nano	1	757779	63134426	2068975	3361863	0.054263	738990499
F_188042	PCR-free	2	852878	64569247	2180018	7934029	0.124367	320396420
		1	709483	51550989	1753305	14425349	0.281215	94143894
F_251175	PCR-free	2	672662	45797284	1605188	6306069	0.139438	196194463
		1	495515	31008533	1115349	8438385	0.274215	56574458
F_198417	PCR-free	2	853900	72155477	2365866	9631180	0.134809	339331281
		1	753758	61720283	2031438	18708110	0.304173	99301743
F_204648	PCR-free	2	514431	40272333	1338091	5120777	0.128122	193936148
		1	380574	30334980	989602	8009966	0.264841	59517624
F_184881	PCR-free	2	603672	42897682	1470562	5369542	0.127079	203711488
		1	467932	28829982	1044640	7264342	0.254274	57132036
F_204510	PCR-free	2	527829	40700253	1373179	5945218	0.147716	149284193
		1	324113	18235037	695701	2922069	0.163692	59486925
F_197773	PCR-free	2	3213575	64690627	4529189	7411979	0.13246	334487318
		1	4291010	52645926	5367162	14004442	0.291582	98076543
F_187464	PCR-free	2	983080	74571529	2552874	9194735	0.125058	370950906
		1	826343	59104941	2051287	15924558	0.270975	112752384
F_184968	PCR-free	2	1837939	110640723	4107239	15000697	0.138978	475982869
		1	1568769	77255006	3138653	20751473	0.272601	140131340
F_182171	PCR-free	2	846844	63186472	2156862	7172435	0.115273	344174477
		1	712446	48567533	1704558	12497390	0.259083	98293631
F_173305	PCR-free	2	904549	59066109	2142933	7660562	0.132085	268343212
		1	741286	40810732	1586048	10850386	0.268928	75798284
F_096887	PCR-free	2	753057	62259844	2029947	9814488	0.158953	211109766
		1	338562	26714113	876818	4406579	0.166428	84146648
**F_078192**	PCR-free	2	648072	46605554	1600276	4852019	0.105393	286878630
		1	1053911	43471190	2016367	10667848	0.247131	82272808
**F_074851**	PCR-free	2	1075079	79548876	2720599	8253526	0.105379	500993586
		1	1427481	74762818	3057529	18081154	0.243812	144112228
**F1_830517**	PCR-free	2	826604	65446108	2192368	8283817	0.127667	330242903
		1	720708	56592447	1879556	16747699	0.296774	94555459
**F1_830043**	PCR-free	1	1308835	96155133	3314643	14935653	0.156796	384114166
**F1_830964**	PCR-free	2	592785	46930900	1569853	6075256	0.130339	232370733
		1	483220	39816019	1295388	11354089	0.285826	70042740
**F1_831241**	PCR-free	2	1557618	114292058	3963642	13635367	0.120982	626917372
		1	2737677	111792696	5264401	32105291	0.289761	174771294
**F1_831536**	PCR-free	2	921352	79958678	2574800	9152029	0.115817	445062716
		1	1199337	68461264	2715665	18366601	0.26993	116091736
**F1_830892**	PCR-free	2	1151356	88028097	2966116	10762510	0.123891	451642355
		1	1022164	73024308	2507784	20468681	0.281729	130908069
**F1_831423**	PCR-free	2	429128	37300868	1214448	4647009	0.125227	188613285
		1	354593	30409425	978823	8038081	0.26486	58653591
Punc	PCR-free	2	1975030	70156271	3671546	8873276	0.132611	323908505
		1	1846933	44658812	2923019	11439503	0.266085	86609202

Supplementary Table 1 – Summary statistics of sequencing and mapping data for each individual. Individuals whose name is in bold are those involved in crossing.

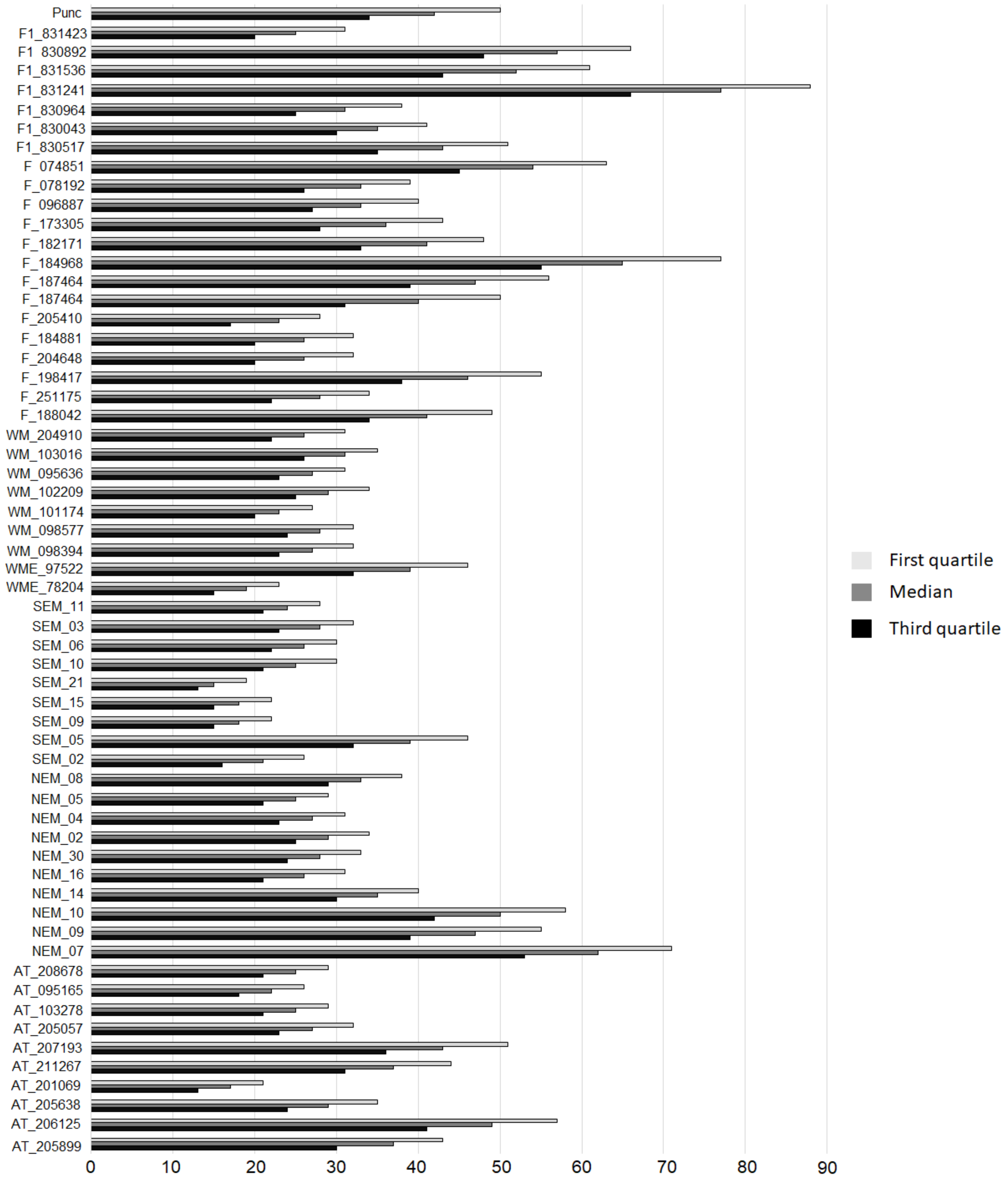

Supplementary Figure 1 - Depth of coverage per individual. Median (dark gray), first (light gray) and third (black) quartile of the depth of coverage for the 10 Atlantic males (AT), the 23 individuals from the western Mediterranean sea (14 females (F) and 9 males (WM/WME)), the 19 individuals from the eastern Mediterranean sea (9 males from the south (SEM) and 9 males from the north (NEM)), the 7 hybrids (F1) and the D. punctatus individual (Punc).

2 – Population structure

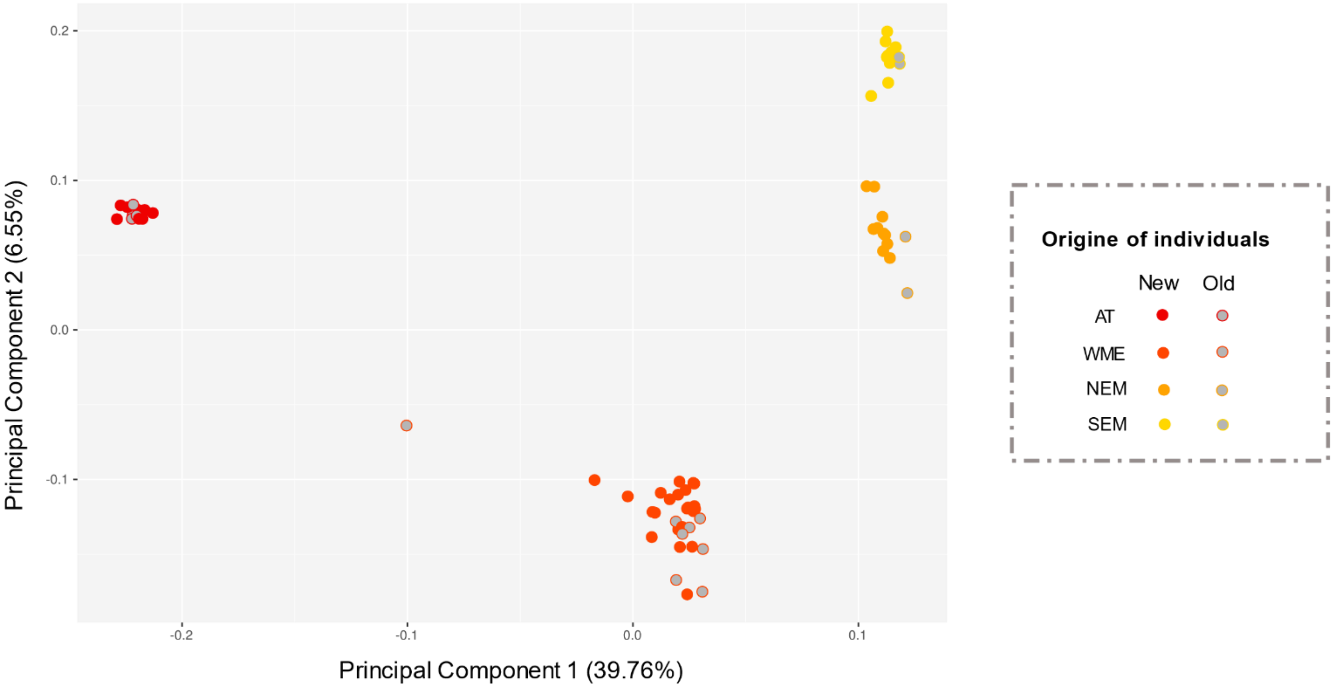

Supplementary Figure 2 - **Principal Component Analysis of the European sea bass population genetic structure.** The analysis was performed on the 52 newly sequenced genomes (colored circles) and the 16 from a previous data set (Duranton et al. 2018) (gray circles with colorful outline). We used the R package adegenet (Jombart 2008) on 91,073 SNPs with a minor allele frequency > 0.4. Individuals originated from four different geographic locations: the Atlantic Ocean (red, AT), the western (orange, WME), northeastern (dark yellow, NEM) and the southeastern (light yellow, SEM) Mediterranean Sea. The first PCA axis explains 39.76% of the total inertia and distinguishes the Atlantic and Mediterranean populations while the second PCA axis explains 6.55% of the total inertia and reveals a substructure within the Mediterranean lineage.

3 – Test for gene flow between *D. labrax* and a third lineage

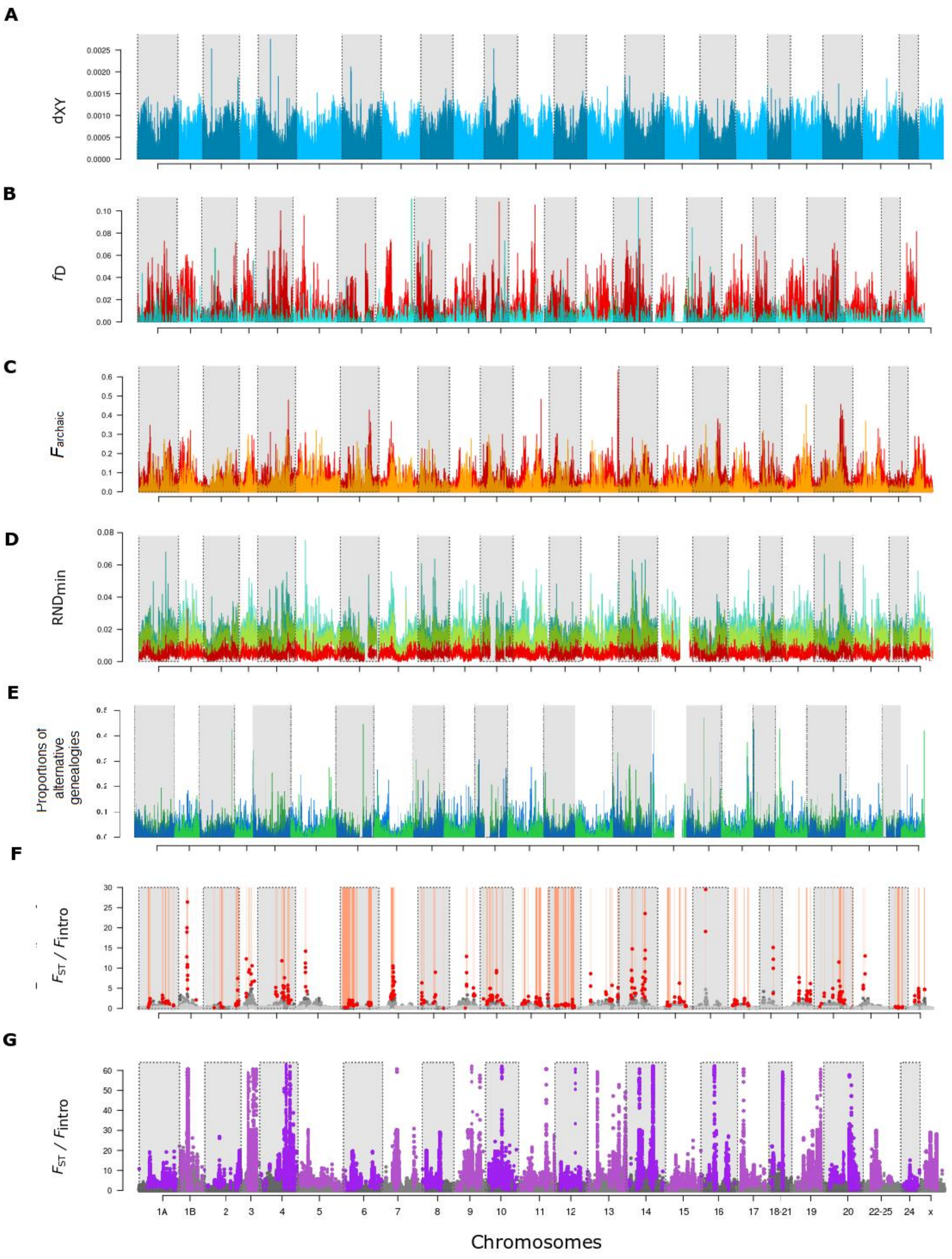

Supplementary Figure 3 – **Statistics measured in non-overlapping 50 kb windows along the genome. A.** d_{XY} measured between the Atlantic and Mediterranean (including eastern and western population) *D. labrax* lineages **B.** f_D statistic measured using in red (((MED, AT), PUN), SAX), in green (((AT, WEST), PUN), SAX) and in blue (((AT, EAST), PUN), SAX). **C.** Fraction of archaic introgressed tracts ($F_{archaic}$) in the eastern Mediterranean and Atlantic population of *D. labrax*. **D.** RND_{min} measured between *D. punctatus* and *D. labrax* Atlantic (red), western (green) and eastern (blue) Mediterranean populations. **E.** Proportion of genealogies grouping *D. punctatus* with the Atlantic (green) or the Mediterranean (blue) *D. labrax* lineage. **F.** Ratio of F_{ST} to F_{intro} used to run the HMM approach on 50 kb windows that rely on 3 states: 1 (light grey), 2 (medium grey) and 3 (dark grey). Red points passed the control for false discovery. We defined islands of reproductive isolation as continuous regions containing only red and dark grey points (red boxes). **G.** Ratio of F_{ST} to F_{intro} used to run the HMM approach on SNPs, purple points are SNPs identified as involved in reproductive isolation that passed the control for false discovery.

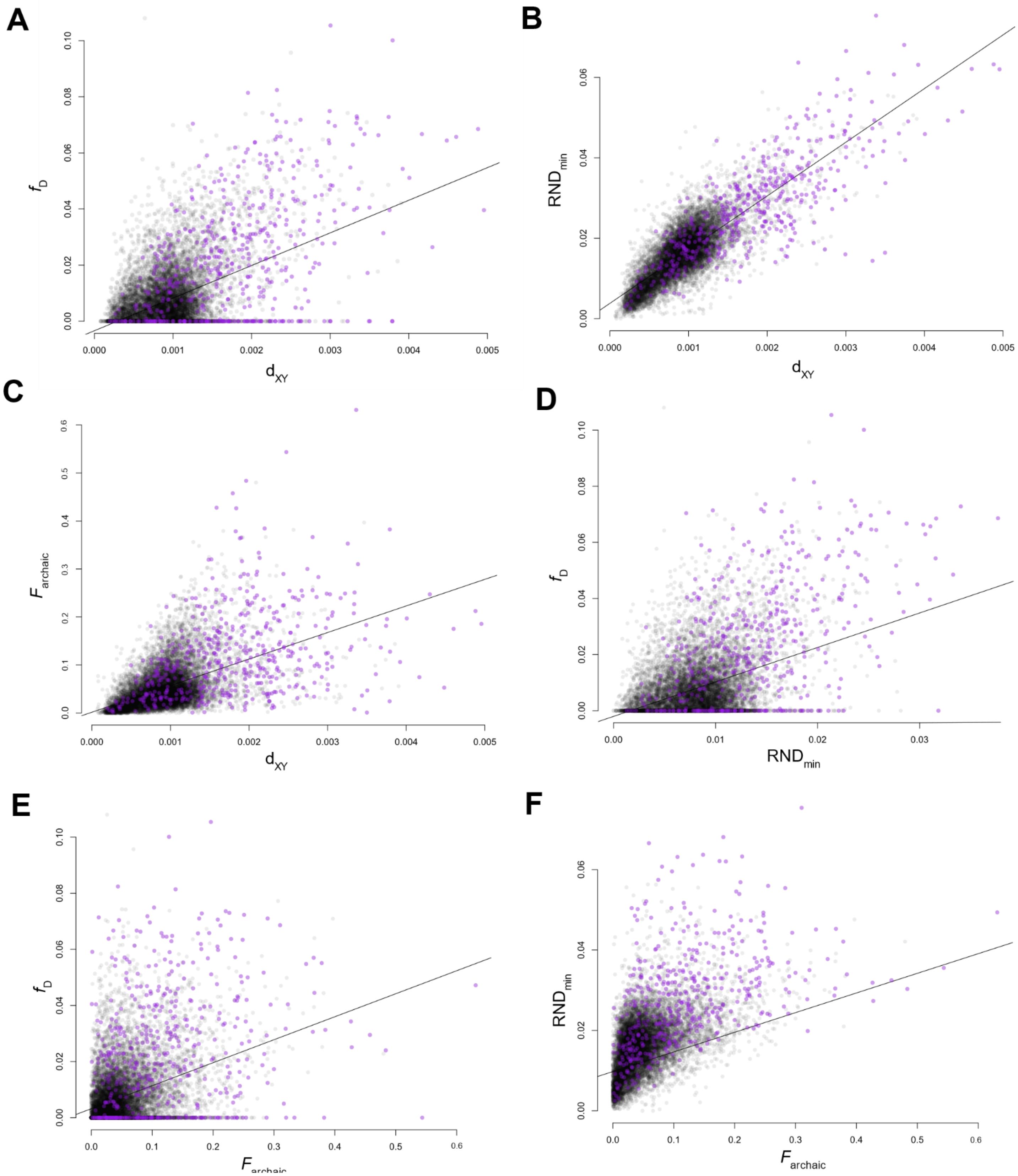

Supplementary Figure 4 – Genome-wide correlations between pairs of statistics measured in non-overlapping 50kb windows. A. f_D (measured using (((MED, AT), PUN), SAX), thus representing gene flow between *D. punctatus* and the *D. labrax* Atlantic lineage) as a function of d_{XY} (measured between the Atlantic and Mediterranean *D. labrax* lineages) **B.** RND_{min} (measured between *D. punctatus* and the Mediterranean *D. labrax* lineage) as a function of d_{XY} **C.** $F_{archaic}$ (inferred in the Atlantic *D. labrax* population) as a function of d_{XY} **D.** f_D as a function of RNDmin **E.** f_D as a function of $F_{archaic}$ and **F.** RND_{min} as a function of $F_{archaic}$. Black lines represent the linear regression line and purple points are windows identified as involved in reproductive isolation with the window-based HMM method.

4 - Detection of introgressed haplotypes

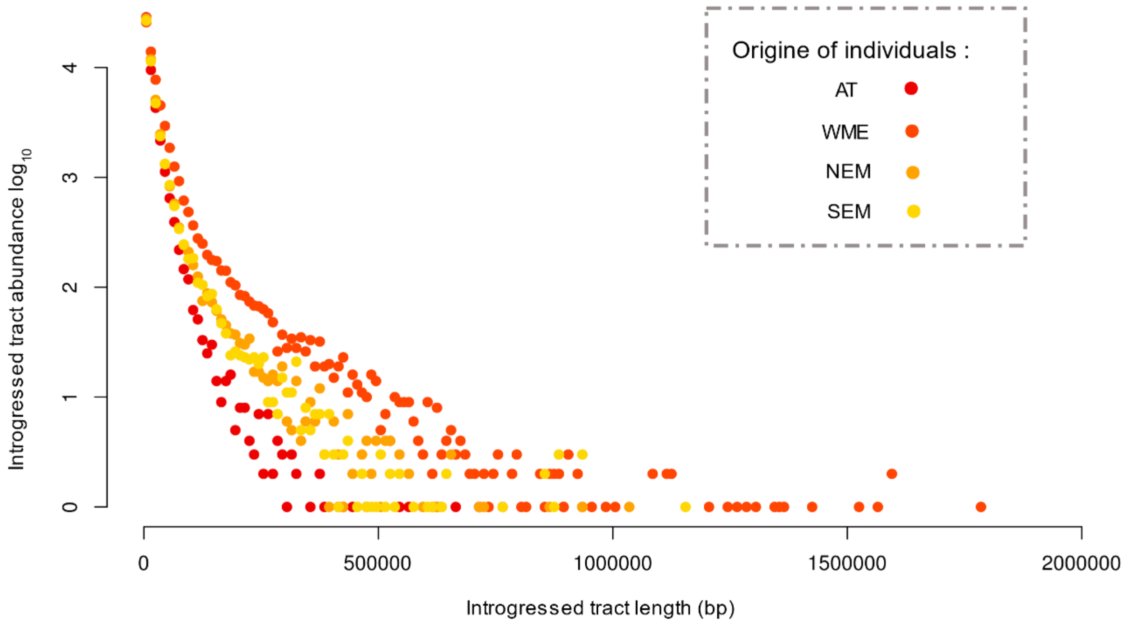

Supplementary Figure 5 – **Introgressed tract length distributions.** Length distributions of Mediterranean tracts introgressed into the Atlantic population (red) and of Atlantic tracts introgressed into the western (orange), northeastern (dark yellow) and southeastern (light yellow) Mediterranean populations. Distributions were generated over the whole genome using 11 individuals per population.

5 - Delineation of regions involved in reproductive isolation between *D. labrax* lineages

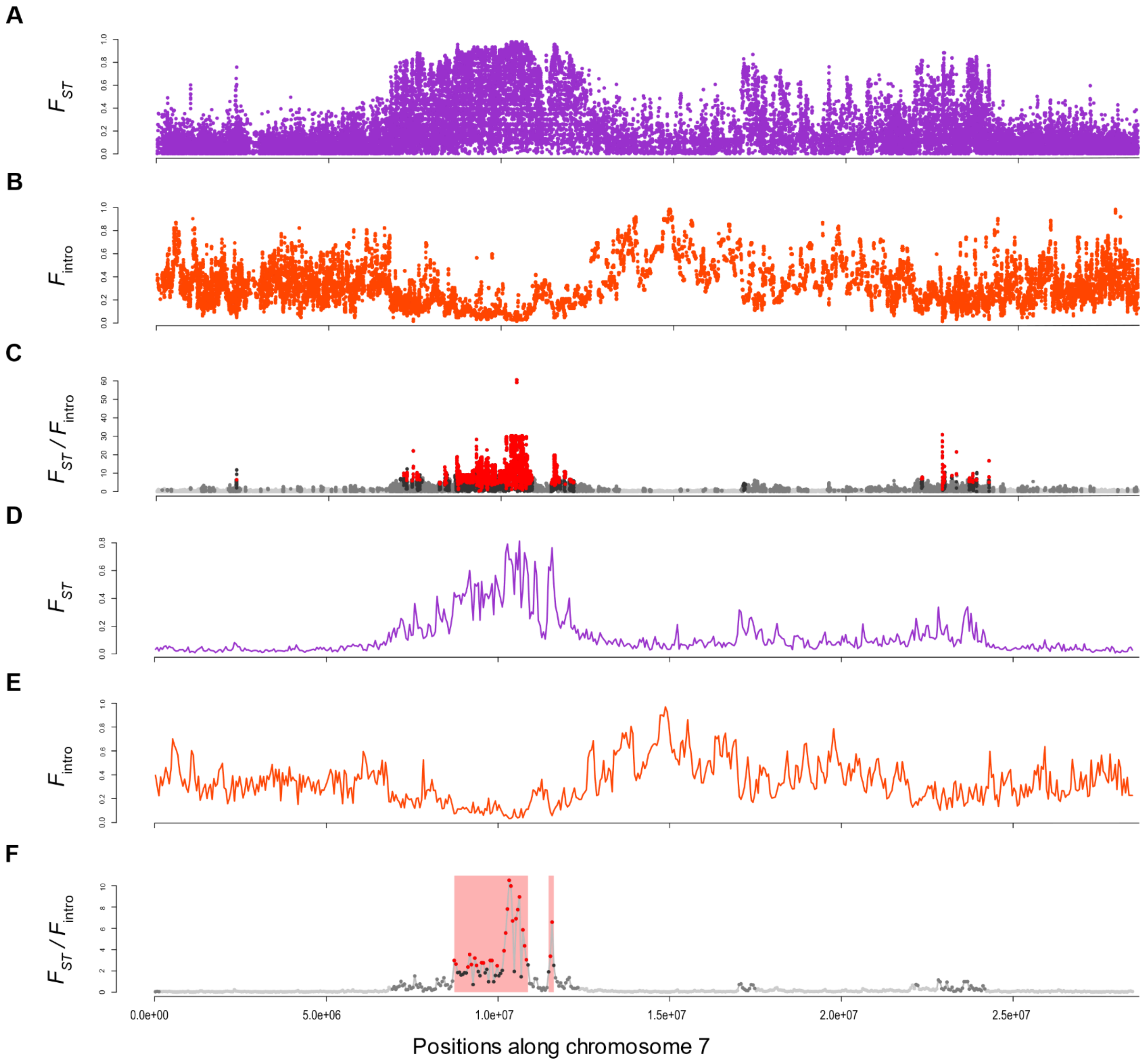

Supplementary Figure 6 – Data and results for the SNPs and 50kb window based HMM approach to identify regions involved in reproductive isolation between the two lineages of *D. labrax* along chromosome 7. A. F_{ST} measured between the Atlantic and western Mediterranean populations of *D. labrax* for each SNPs and in every non-overlapping 50 kb window (**D**). **B.** Fraction of Atlantic tracts introgressed in western Mediterranean genomes (F_{intro}) for each SNPs and in every non-overlapping 50 kb windows (**E**). **C.** Statistic analyzed by the HMM approach (F_{ST} divided by F_{intro}) for each SNPs and every 50 kb non-overlapping window (**F**). Ratio of F_{ST} to F_{intro} used to run the HMM approach that relies on 3 states to identify: neutral genomic regions (state 1, light grey), genomic regions under linked selection (state 2, medium grey) and genomic regions involved in reproductive isolation (state 3, dark grey). Red points are those that passed the control for false discovery. For the window approach we defined island of RI as continuous regions containing only red and dark grey points (red boxes).

6 – Frequency of introgressed *D. punctatus* tracts

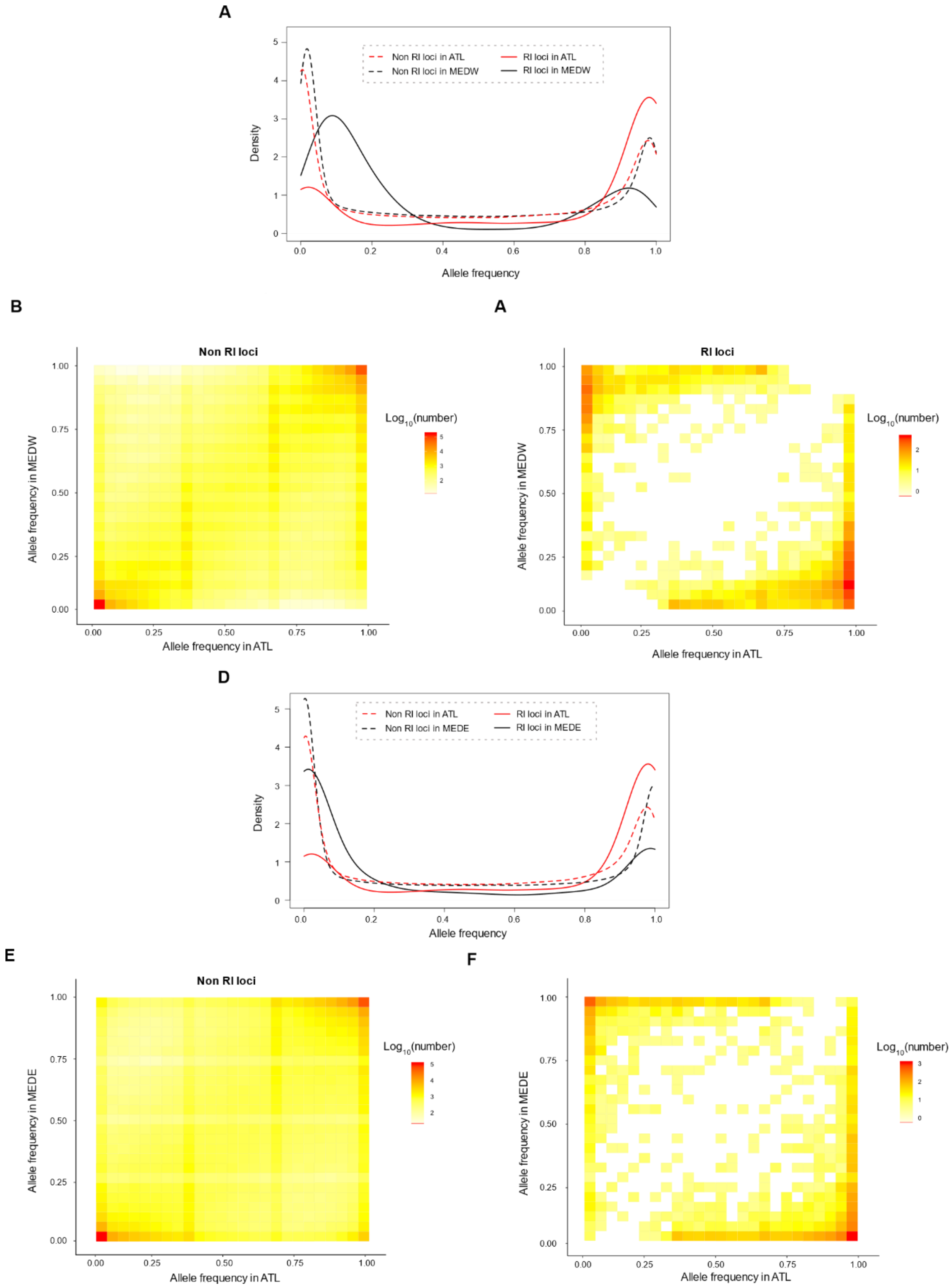

Supplementary Figure 7 – **Distributions and joint allele-frequency spectrums of derived *D. punctatus* alleles present in *D. labrax*.** Distribution of *D. punctatus* derived alleles frequency in Atlantic (red) and western (black) (**A**) or eastern (**D**) Mediterranean *D. labrax* individuals for loci involved (solid line) or not (dashed lines) in reproductive isolation between the two *D. labrax* lineages. **B.** Joint allele-frequency spectrum of derived *D. punctatus* alleles for the western Mediterranean (31 individuals) and Atlantic (14 individuals) populations for 594,797 SNPs not involved and 7,372 SNPs involved (**C**) in reproductive isolation. **E.** Joint allele-frequency spectrum of derived *D. punctatus* alleles for the eastern Mediterranean (23 individuals) and Atlantic (14 individuals) populations for 594,454 SNPs not involved and 7,366 SNPs involved (**C**) in reproductive isolation.

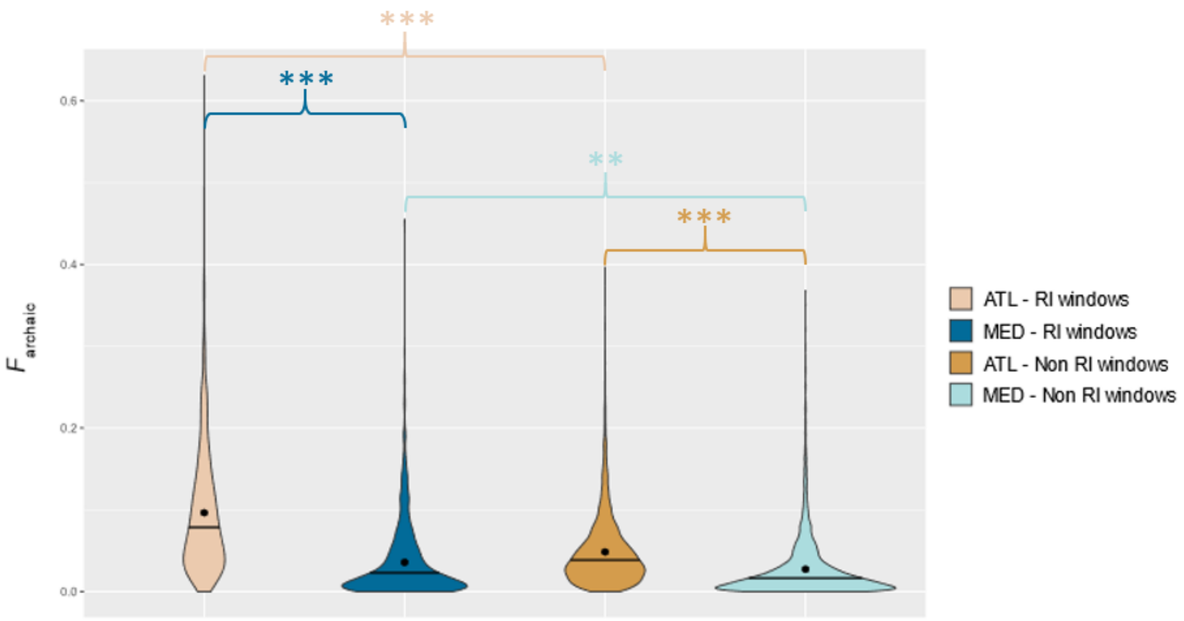

Supplementary Figure 8 – Distributions of the fraction of archaic tracts for Atlantic and Mediterranean genomes inside and outside regions involved in RI. Black horizontal line represents the median and the black point the mean of each distribution.

7 – Estimation of the time since introgression between *D. punctatus* and *D. labrax*

Chromosome	*P*	*r*	*a*	T_{admix}
1A	0.062	$2.22e^{-8}$	0.063	94499
1B	0.044	$5.62e^{-8}$	0.060	66152
2	0.037	$4.55e^{-8}$	0.038	105730
3	0.048	$3.25e^{-8}$	0.053	139364
4	0.047	$4.68e^{-8}$	0.058	87390
5	0.047	$4.44e^{-8}$	0.056	93722
6	0.048	$4.38e^{-8}$	0.052	105456
7	0.055	$3.29e^{-8}$	0.051	161342
8	0.051	$4.72e^{-8}$	0.041	131003
9	0.041	$5.55e^{-8}$	0.042	86450
10	0.061	$4.69e^{-8}$	0.058	112733
11	0.046	$4.79e^{-8}$	0.060	79832
12	0.058	$4.39e^{-8}$	0.055	120243
13	0.045	$4.05e^{-8}$	0.058	95632
14	0.061	$5.33e^{-8}$	0.052	110692
15	0.048	$6.08e^{-8}$	0.049	81369
16	0.048	$5.08e^{-8}$	0.057	82769
17	0.048	$4.75e^{-8}$	0.044	114928
18-21	0.059	$3.75e^{-8}$	0.064	123309
19	0.038	$4.85e^{-8}$	0.054	72489
20	0.045	$4.30e^{-8}$	0.058	90803
22-25	0.057	$4.89e^{-8}$	0.057	102440
24	0.082	$9.38e^{-8}$	0.048	90731
X	0.066	$5.63e^{-8}$	0.064	90680

Supplementary Table 2 – values used to estimate T_{admix} for each chromosome.

8 – References

Duranton M., F. Allal, C. Fraïsse, N. Bierne, F. Bonhomme, *et al.*, 2018 The origin and remolding of genomic islands of differentiation in the European sea bass. Nature Communications 9: 2518. https://doi.org/10.1038/s41467-018-04963-6

Jombart T., 2008 adegenet: a R package for the multivariate analysis of genetic markers. Bioinformatics 24: 1403–1405. https://doi.org/10.1093/bioinformatics/btn129
